# Asthma in Sickle Cell Disease

**DOI:** 10.1100/tsw.2011.105

**Published:** 2011-05-26

**Authors:** Manisha Newaskar, Karen A. Hardy, Claudia R. Morris

**Affiliations:** ^1^ Bay Area Pediatric Pulmonary Medical Corporation Children's Hospital & Research Center at Oakland Oakland CA; ^2^ Department of Emergency Medicine Children's Hospital & Research Center at Oakland Oakland CA

**Keywords:** sickle cell disease, asthma, obstructive lung disease, airway hyper-reactivity, proinflammatory state, arginase, nitric oxide, arginine metabolome, asthma therapy

## Abstract

In recent years, evidence has increased that asthma predisposes to complications of sickle cell disease (SCD), such as pain crises, acute chest syndrome, pulmonary hypertension, and stroke, and is associated with increased mortality. An obstructive pattern of pulmonary function, along with a higher-than-expected prevalence of airway hyper-responsiveness (AHR) when compared to the general population, has led some researchers to suspect that underlying hemolysis may contribute to the development of a pulmonary disease similar to asthma in patients with SCD. While the pathophysiologic mechanism in atopic asthma involves up-regulation of Th2 cytokines, mast cell– and eosinophil-driven inflammation, plus increased activity of inducible nitric oxide synthase (iNOS) and arginase in airway epithelium resulting in obstructive changes and AHR, the exact mechanisms of AHR, obstructive and restrictive lung disease in SCD is unclear. It is known that SCD is associated with a proinflammatory state and an enhanced inflammatory response is seen during vaso-occlusive events (VOE). Hemolysis-driven acute-on-chronic inflammation and dysregulated arginines–nitric oxide metabolism are potential mechanisms by which pulmonary dysfunction could occur in patients with SCD. In patients with a genetic predisposition of atopic asthma, these changes are probably more severe and result in increased susceptibility to sickle cell complications. Early recognition and aggressive management of asthma based on established National Institutes of Health asthma guidelines is recommended in order to minimize morbidity and mortality.

## INTRODUCTION

Asthma is a common comorbid factor in sickle cell disease (SCD). However, the incidence of asthma in SCD is much higher than expected compared to rates in the general population. Whether "asthma" in SCD is purely related to genetic and environmental factors, or rather is the consequence of the underlying hemolytic state, is a topic of recent debate. In this article, we will review the available literature on the role of asthma in SCD, possible pathophysiologic mechanisms for the development of asthma in SCD, and proposed management strategies.

## Prevalence of Asthma and Lung Function Abnormality in SCD

About 8 in 100 individuals in the U.S. suffer from asthma currently, according to a Centers for Disease Control and Prevention report in 2008. The lifetime prevalence of asthma in children is estimated to be 13.8%, whereas in African-American children, lifetime asthma prevalence is much higher at around 21%[1]. It is estimated that about 30–70% of patients with SCD have asthma. This observation comes from several studies that have reported an increased prevalence of lower airway obstruction and airway hyper-reactivity (AHR) in children with SCD. In a cross-sectional study, Koumbourlis et al. reported a 35% prevalence of lower airway obstruction in 5- to 18-year-old children with a homozygous HbSS mutation. A large number of subjects (approximately 54%), regardless of their lung function status (normal vs. obstructive or restrictive), showed a positive bronchodilator response on pulmonary function testing, suggesting an increased prevalence of AHR in this population[2]. Leong et al. reported a prevalence of 73% for AHR by cold air challenge, regardless of asthma symptoms in SCD patients, and felt that there may be an alternate mechanism of AHR in SCD[3]. Subsequently, many recent studies have shown a similar prevalence of AHR (range: 48–77%) as measured by the methacholine challenge test in patients with SCD[4,5,6,7]. Prevalence of nonspecific AHR has been reported to be around 20% in the pediatric population[8]. Strunk et al. demonstrated that children with SCD and clinical symptoms of asthma who had normal results of spirometry, including negative bronchodilator response, had positive results to the methacholine challenge test[6]. The majority of children in this study had evidence of aeroallergen sensitivity, suggesting typical asthma. However, this study only included children with asthma-like symptoms and significant morbidity related to their SCD. In a more recent multicenter study, Field et al. studied response to methacholine challenge in patients with SCD, regardless of their asthma history, and found no relationship between AHR and allergic diathesis other than elevated serum IgE levels[7]. In fact, serum lactate dehydrogenase (LDH), a biomarker of hemolysis[9], was associated with increased AHR, implicating hemolysis as a potential causative factor for AHR in SCD[7].

## Increased Rate of Complications in Patients with Asthma and SCD

Asthma in SCD is known to be associated with increased morbidity and an elevated rate of sickle cell complications, such as acute chest syndrome (ACS), stroke, vaso-occlusive episodes (VOE), and early mortality[10,11,12,13,14,15]. One study found a fourfold increased likelihood of the development of ACS in the presence of physician-diagnosed asthma history in hospitalized patients with SCD[11]. In a large prospective study by Boyd et al., children with SCD and a clinical diagnosis of asthma had nearly twice as many ACS episodes (0.39 vs. 0.20 events per patient year) and increased VOE (1.49 vs. 0.37 events per patient year) when compared to children without asthma, even after controlling for previously identifiable risk factors[12]. In a case control study, Knight-Madden et al. found that children with SCD who had suffered from recurrent episodes of ACS were more likely to have atopic asthma compared to children who had no prior history of ACS or only a single episode of ACS[10]. This study also found an increased prevalence of bronchial hyper-reactivity in SCD compared to ethnically matched controls, but there was no relationship between the presence of bronchial hyper-responsiveness and incidence of ACS. Interestingly, the prevalence of atopy was similar in the SCD and control groups, suggesting that in patients with SCD, the mechanism of "asthma" was different from that seen in classic allergic asthma.

It is postulated that repeated episodes of ACS lead to sickle cell chronic lung disease. Hagar et al. found an association between asthma or obstructive lung disease and an increased risk for pulmonary hypertension in children with SCD[16]. In an analysis of a prospective cohort of individuals enrolled in the cooperative study for SCD, subjects with a diagnosis of asthma had an over twofold mortality risk compared to subjects without a diagnosis of asthma[15]. The median life span for individuals with SCD and asthma was 52.5 vs. 64.3 years of age for individuals without asthma. Thus, asthma seems to be an important risk factor for premature death in patients with SCD. Early diagnosis and aggressive management is therefore necessary to prevent asthma-related morbidity and mortality in SCD.

## Pathophysiology of Asthma

According to the National Asthma Education and Prevention Program (NAEPP) Expert Panel Report (EPR), the working definition of asthma is as follows: Asthma is a chronic inflammatory disorder of the airways in which many cells and cellular elements play a role: in particular, mast cells, eosinophils, T lymphocytes, macrophages, neutrophils, and epithelial cells. In susceptible individuals, this inflammation causes recurrent episodes of wheezing, breathlessness, chest tightness, and coughing, particularly at night or in the early morning. These episodes are usually associated with widespread, but variable, airflow obstruction that is often reversible, either spontaneously or with treatment. The inflammation also causes an associated increase in the existing bronchial hyper-responsiveness to a variety of stimuli. Reversibility of airflow limitation may be incomplete in some patients with asthma[17].

Asthma in the general population is a complex and multifactorial disorder influenced by genetic polymorphisms, as well as environmental and infectious triggers. Not all patients with "asthma" have the same disease paradigm even though the clinical manifestations of asthma may be similar. Just as "anemia" is a clinical diagnosis that does not designate a mechanistic etiology and can be induced by a variety of diverse triggers, from iron, folate, or B12 deficiency to acute hemorrhage, "asthma" is a clinical diagnosis that denotes airway inflammation and bronchial reactivity induced by diverse triggers and inflammatory pathways that may vary from one individual to the next. This may be a fundamental concept when considering the "asthma-like" condition commonly found in SCD.

## AIRWAY INFLAMMATION IN ASTHMA

Complex interactions between various cells and inflammatory mediators are seen in asthma. Classically, asthmatic airways show a CD4+ lymphocyte–, eosinophil-, and macrophage-rich inflammatory response. There is a predominance of Th2 cytokines (including interleukins [IL-4, -5, and -13]), and up-regulation of chemokines (including regulated on activation, normal T-cell–expressed and secreted [RANTES]), eotaxins, and monocyte chemoattractant protein-1[18,19]. These cytokines and chemokines cause influx of eosinophils in the airway matrix, which in turn release inflammatory mediators, such as leukotrienes and granule proteins that cause airway inflammation. Activation of mucosal mast cells releases histamine, cysteinyl-leukotrienes (CysLT), and prostaglandin D2, which cause bronchoconstriction. Leukotrienes are derived from arachidonic acid via the 5-lipoxygenase pathway. CysLT (LTC4, LTD4, and LTE4) are potent bronchoconstrictors, whereas LTB4 is a chemoattractant that causes recruitment of neutrophils and prolongs the inflammatory response[20,21]. IgE is the antibody responsible for activation of allergic reactions and plays an important role in the pathophysiology of allergic asthma. Mast cells have a large number of high-affinity IgE receptors and when activated by an antigen, release a wide variety of mediators to initiate acute bronchospasm and also release proinflammatory cytokines to perpetuate underlying airway inflammation[22,23]. Neutrophil-dominated inflammation has been described in severe asthma exacerbations[24].

## Role of Arginine–Nitric Oxide Metabolism in Asthma

Alterations in L-arginine and nitric oxide (NO) metabolism in the lung have been shown to play a role in asthma pathophysiology[25,26,27]. L-arginine, a semi-essential amino acid, is an important common substrate for both the arginases and nitric oxide synthase (NOS) enzyme families. NO is an important vasodilator of the bronchial circulation, with both bronchodilatory and anti-inflammatory properties, and is synthesized from oxidation of its obligate substrate L-arginine, which is catalyzed by a family of NOS enzymes. Three isoforms of NOS are recognized: NOS I, II, and III. NOS I is constitutively expressed in the airway epithelium and inhibitory nonadrenergic noncholinergic (iNANC) neurons, while NOS III is expressed in airway vasculature endothelial cells and airway epithelium. Their activity is regulated by intracellular calcium, with rapid onset of activity and production of small amounts of NO. NOS II or inducible NOS (iNOS) is transcriptionally regulated by proinflammatory stimuli, with the ability to produce large amounts in NO over a few hours[28,29]. In asthmatic patients as well as experimental models of asthma, increased NO production occurs in the airways related to up-regulation of NOS II (iNOS) by proinflammatory cytokines after allergen challenge and during the late asthmatic reaction[28]. This up-regulation of NOS II in airway epithelial cells and inflammatory cells is associated with airway eosinophilia, AHR, and increased NO in exhaled air[30,31]. The increased production of NO itself may not be responsible for AHR, as NO seems to have a protective effect on bronchial muscle tone. It is believed that the AHR after the late asthmatic reaction is caused by increased formation of peroxynitrite[32] that occurs due to reduced availability of L-arginine for NOS II, which potentially causes uncoupling of this enzyme[33]. Increased activity of the arginase enzyme, which competes with NOS for the substrate L-arginine, seems to be, at least in part, responsible for this process[34].

Arginase is an essential enzyme in the urea cycle, responsible for the conversion of arginine to ornithine and urea. The NOS and arginase enzymes can be expressed simultaneously under a wide variety of inflammatory conditions, resulting in competition for their common substrate[25]. Two forms of arginase have been identified: type 1, a cytosolic enzyme highly expressed in the liver, and type 2, a mitochondrial enzyme found predominantly in the kidney, prostate, testis, and small intestine[25]. Both forms are expressed in human airways. Arginase-1 is also present in human red blood cells[35,36,37], which has significant implications for hemolytic disorders[38].

In mice models of asthma, arginase activity is noted to be increased in allergen-challenged lungs[39]. Increased arginase I activity has been demonstrated in inflammatory cells and airway epithelium from bronchial biopsies as well as bronchoalveolar lavage (BAL) samples from asthmatic patients[39]. Further, increases in serum arginase, along with reduction in plasma L-arginine levels, is seen in asthmatic patients experiencing an acute exacerbation[25]. As both arginase and NOS use arginine as a common substrate, arginase decreases the bioavailability of L-arginine for NOS, thereby limiting NO production and subsequently may affect airway tone and inflammation. Arginase may also play a role in the development of chronic airway remodeling through formation of L-ornithine with downstream production of polyamines and L-proline, which are involved in processes of cellular proliferation and collagen deposition (Fig. 1).

## PATHOPHYSIOLOGY OF PULMONARY INFLAMMATION IN SCD

Asthma in SCD may be a very different entity with regards to the underlying pathophysiologic mechanism and clinical expression. However, there are many inflammatory pathways common to both disorders that may set the stage for an asthma-like condition in SCD. It is likely that genetic predisposition and the classic eosinophil- and macrophage-rich inflammation, as described in the previous section, occurs in patients with SCD who clearly have evidence of allergic diathesis and familial asthma. In a very recent report of death due to asthma in two adolescents with SCD, autopsy findings showed smooth muscle hyperplasia and eosinophilia consistent with classic asthma[40]. However, it is also plausible that hemolysis itself induces an inflammatory state in the lungs and airways of patients with SCD, contributing to changes in lung function and bronchial hyper-reactivity[38].

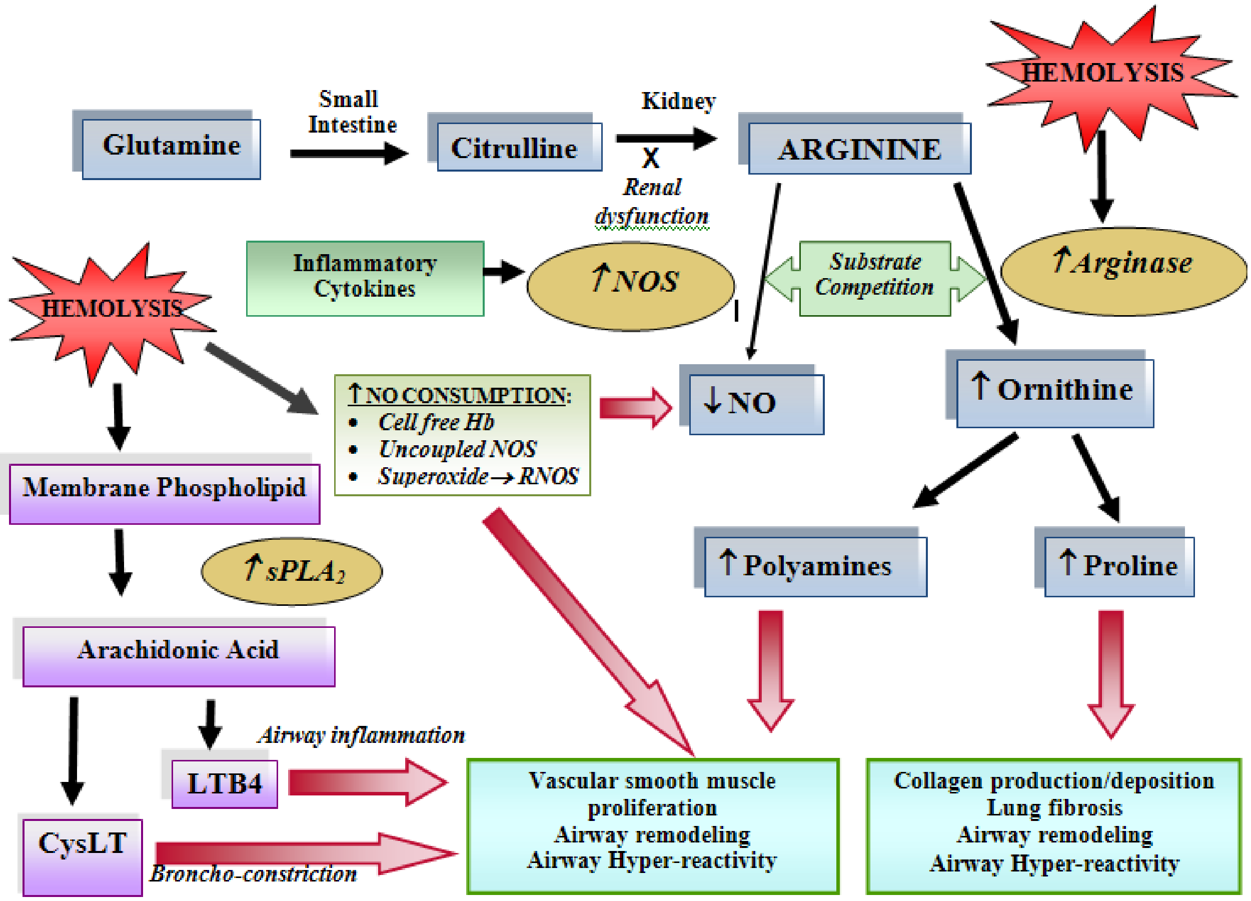

**FIGURE 1.** Novel paradigm of hemolysis-associated AHR and an “asthma-like” condition in SCD. Dietary glutamine serves as a precursor for the *de novo* production of arginine through the citrulline-arginine pathway. Arginine is synthesized endogenously from citrulline primarily via the intestinal-renal axis. Arginase and NOS compete for arginine, their common substrate. In SCD, bioavailability of arginine and NO is decreased by several mechanisms linked to hemolysis. The release of erythrocyte arginase during hemolysis increases plasma arginase levels and shifts arginine metabolism toward ornithine production, limiting the amount of substrate available for NO synthesis. The bioavailability of arginine is further diminished by increased ornithine levels because ornithine and arginine compete for the same transporter system for cellular uptake[57]. Despite increases in NOS, NO bioavailability is low due to low substrate availability[57], NO scavenging by cell-free hemoglobin released during hemolysis[99] NOS uncoupling[100], and through reactions with free radicals, such as superoxide and other reactive NO species[101]. Superoxide reacts with NO to form reactive NO species (RNOS), including peroxynitrite, which can contribute further to cell damage and cell death[64]. Endothelial dysfunction resulting from NO depletion and increased levels of the downstream products of ornithine metabolism (polyamines and proline) likely contribute to the pathogenesis of lung injury, asthma, and pulmonary hypertension in SCD[65]. As a result of hemolysis, membrane phospholipids are hydrolyzed by secretory phospholipase A2 (sPLA2) into arachidonic acid (AA). AA metabolism via the 5-lipoxygenase pathway leads to formation of CysLT and LTB4. LTB4 is an important chemoattractant of neutrophils and plays an important role in airway inflammation[53]. In airways, CysLT cause bronchoconstriction[56], smooth muscle proliferation, airway edema, and mucus production[55], whereas in vasculature, they cause vasoconstriction, vascular leakage, and up-regulation of cellular adhesion molecules. (Modified with permission from the American Society of Hematology[65].)

## Enhanced Inflammation in SCD

Similar to asthma, SCD is considered to be a proinflammatory state. The plasma levels of the proinflammatory cytokines IL-1β, IL-6, interferon (IFN)-γ, and tumor necrosis factor (TNF)-α are noted to be elevated at steady state, with further increases during acute VOE[41]. Vascular adhesion molecule-1 (VCAM-1) is also shown to be elevated at baseline, with additional elevations during VOE and ACS[42], while arginine and NO bioavailability drop acutely[43]. There is evidence that infectious and allergic triggers lead to enhanced pulmonary inflammation in SCD. Also, hypoxia-reperfusion injury sets off an inflammatory cascade in the lungs, which produces further pulmonary dysfunction.

Holtzclaw et al. found that in sickle cell mice, endotoxin lipopolysaccharide (LPS) challenge is associated with a significant increase in mortality ($p < 0.005$) and airway tone ($p < 0.02$). Serum and BAL fluid levels of cytokines TNF-α, IL-1β, and sVCAM-1 were increased after LPS challenge in sickle cell mice compared with controls, suggesting that an enhanced response to an inflammatory insult could play a role in the increased susceptibility to pulmonary dysfunction[44]. Interestingly, increases in airway tone in SCD mice were not associated with the presence of inflammatory cells in bronchioles or airway smooth muscle despite an increase in BAL cytokine levels, suggesting an alternate mechanism of airway obstruction in SCD. Nandedkar et al. experimentally induced asthma in sickle cell mice by sensitization to ova-albumin and found increased mortality compared to unsensitized mice[45]. Sickle cell mice demonstrated an exaggerated response to allergen-induced lung inflammation, further supporting the observation that an enhanced inflammatory response is seen in SCD. A recent study by Wallace et al. showed a role for CD1d-restricted invariant natural killer T cells (iNKT) in causing chronic pulmonary inflammation and dysfunction in mice and possibly in humans with SCD. iNKT cells were noted to be increased in number and activation in the lung at a baseline steady state, and further increased after hypoxia/reoxygenation injury, triggering an inflammatory cascade via the IFN- γ–inducible chemokines-CXCR3 axis, resulting in increased vascular permeability and decreased arterial oxygen saturation[46].

Based on the results of these studies, it could be said that in patients with SCD, any viral or bacterial infection or allergic triggers induce heightened inflammation on top of an underlying inflammatory state, which could result in pulmonary dysfunction. Similarly, repeated nocturnal hypoxia from sleep-disordered breathing could lead to hypoxic injury and trigger an inflammatory cascade, leading to further sickle cell complications.

## Role of Leukotrienes in SCD

Recent studies have shown a potential role for leukotrienes in the pathogenesis of VOE and ACS[47,48,49]. Leukotrienes are inflammatory mediators produced from arachidonic acid (AA) via the 5-lipoxygenase pathway. AA is derived from membrane phospholipids through hydrolysis by secretory phospholipase A2 (sPLA2), an essential step to leukotriene generation. sPLA2 levels increase acutely during ACS and VOE[50]. In one study, red blood cell transfusion was found to prevent ACS predicted by elevated sPLA2 compared to the 63% of patients with an elevated sPLA2 randomized to standard care (no transfusion) who went on to develop ACS[51]. There also are data to suggest a role for elevated sPLA2 in asthma in general[52], which is of interest given the difficulty in clinically differentiating ACS from asthma in a patient with SCD. Leukotrienes, especially LTB4, and CysLT levels are also increased in patients with SCD at baseline and further increased during VOE. LTB4, which causes neutrophil activation and chemotaxis, possibly contributes to the process of vaso-occlusion[53]. In a prospective study, Setty and Stuart demonstrated that LTB4 is increased in subjects with SCD compared to controls without SCD[54]. CysLT play an important role in pathogenesis of asthma and have effects on airways and vasculature. In airways, CysLT cause bronchoconstriction, smooth muscle proliferation, airway edema, and mucus production, whereas in vasculature, they cause vasoconstriction, vascular leakage, and up-regulation of cellular adhesion molecules[21,55,56]. Jennings and associates, in a cross-sectional observational study, found that the urinary level of LTE4, which is a metabolite of CysLT, was significantly higher at baseline in SCD children with or without diagnosis of asthma compared to healthy controls[49]. Later, Field and associates, in a prospective study, found that CysLT levels increased significantly from baseline during painful episodes, suggesting a potential role of CysLT in VOE[48]. Furthermore, higher baseline levels and a greater increase in LTE4 from baseline to pain state was seen in individuals with higher rates of hospitalization for pain, thus suggesting polymorphisms in the CysLT gene contributing to a variable phenotype. These data show that leukotrienes play an important role in the pathogenesis of VOE and ACS, and may be implicated in the pathogenesis of lung disease/asthma in SCD (Fig. 1).

## Role of Arginine–Nitric Oxide Metabolism in SCD

As discussed above, alterations in the L-arginine–NO pathway in the lung have been shown to play a role in asthma pathophysiology. Likewise, there is growing evidence that dysregulated NO and arginine metabolism leads to complications of SCD[57]. Patients with SCD are arginine deficient at baseline, while plasma levels drop further during ACS and VOE[43]. Low arginine bioavailability is also associated with early mortality[57]. In SCD, bioavailability of both arginine and NO is decreased by several mechanisms linked to hemolysis[57,58]. Plasma arginase activity is elevated in SCD as a consequence of inflammation, liver dysfunction, and, most significantly, by the release of erythrocyte arginase during intravascular hemolysis, which has been demonstrated by the strong correlation between plasma arginase levels and cell-free hemoglobin levels and other markers of increased hemolytic rate, including LDH[57]. However, the impact of excess arginase in the circulation of patients with SCD on "asthma" has not yet been investigated.

It is unclear if similar alterations in NO production occur in the airway epithelium of SCD patients as seen in allergic asthma. It is well known that fractional exhaled NO (FeNO) levels are increased in patients with asthma and is considered a surrogate for eosinophilic airway inflammation, which is presumed to originate from increased NOS II expression in the respiratory epithelial cells[59]. In contrast, FeNO levels are reported to be low in patients with SCD[60,61], which is not clearly understood so far. It is likely that increased consumption of NO occurs in SCD due to the presence of increased oxidative stress[62,63], as NO, being a labile molecule, easily reacts with $O_2$ radicals, causing formation of peroxynitrite. Also, neuronal NOS can generate peroxynitrite in the presence of an L-arginine deficiency[64].

For a variety of reasons, SCD patients with a higher hemolytic rate (as indicated by higher LDH[9]) likely have a lower arginine and NO bioavailability compared to those with lower rates of hemolysis at baseline[65]. Low NO bioavailability may contribute to increased neuronal bronchoconstriction, pulmonary vasoconstriction, and changes that translate to pulmonary hypertension and an asthma-like state. Interestingly, in a recent prospective cohort study, Field et al.[7] reported no relationship of methacholine-induced AHR with asthma symptoms or physician diagnosis of asthma. However, AHR was noted to be related to increased LDH levels, suggesting hemolysis as a potential mechanism contributing to AHR and asthma in SCD. Hagar and colleagues[16] found an association of asthma in children with SCD who had an elevated tricuspid regurgitant jet velocity on Doppler echocardiography, a measure that is strongly associated with hemolytic rate[66,67]. Of interest, this association was not found in adults with SCD[16]. Future studies are needed in order to validate this hypothesis that includes asthma[68] in the hemolytic subphenotype of SCD[69] (Fig. 2).

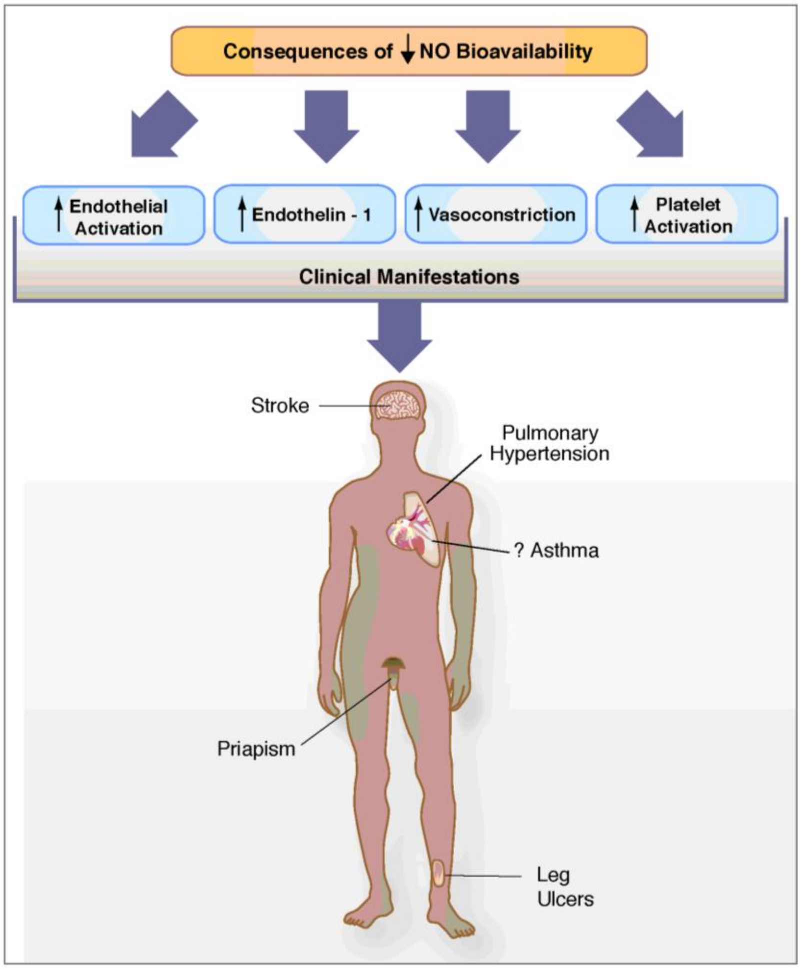

**FIGURE 2.** Consequences of low NO bioavailability. The consequences of decreased NO bioavailability include endothelial cell activation, up-regulation of the potent vasoconstrictor endothelin-1, vasoconstriction, platelet activation, increased tissue factor, and activation of coagulation pathways, all of which ultimately translate into the clinical manifestations of SCD. NO bioavailability is particularly vulnerable to the effects of hemolysis, an event in SCD that contributes to the development of the hemolytic subphenotypes[69], which include pulmonary hypertension, priapism, cutaneous leg ulceration, stroke, and possibly asthma. (Reproduced with permission from the American Society of Hematology[65].)

## DIAGNOSING ASTHMA IN SCD

### Past Medical and Familial History

There is evidence of asthma as a separate comorbid condition in patients with SCD. Phillips et al. reported a familial pattern of inheritance of asthma among first-degree relatives of probands with SCD and asthma[70]. Field and colleagues found a sibling history of asthma as a risk factor for pain crisis in children with SCD, suggesting disease-modifying effects of asthma due to familial factors[71]. Since parental history of asthma was not associated with a significantly increased risk of pain or ACS episodes in this study, it is not clear if the association seen with sibling history was purely genetic or due to environmental influences. Polymorphisms in the NOS gene have been reported to be associated with an increased risk of ACS[72,73,74]. Duckworth et al.[72] reported an AAT repeat polymorphism in the NOS I gene to be associated with risk of ACS in individuals without physician-diagnosed asthma, implicating a genetic basis for disease. However, due to the small number of subjects, this finding should be interpreted with caution.

A detailed history of pulmonary symptoms and careful review of systems at each clinic visit should be undertaken in order to recognize symptoms of asthma or pulmonary disease in this population. It is not clear if clinical features of asthma are similar in patients with SCD compared to the general population. Typical features of allergic sensitization and atopic dermatitis, if present along with a history of wheezing/chronic cough, should prompt a physician to consider the diagnosis of asthma and treat aggressively to minimize complications of SCD. Even children with no clear-cut history of pulmonary/asthma symptoms should undergo pulmonary function evaluation given the high prevalence of abnormal pulmonary function in this population[2,12].

The role of environmental tobacco smoke exposure should not be discounted when assessing patients with repeated respiratory exacerbations/wheezing problems. History of snoring, daytime somnolence, and presence of tonsillar and adenoidal hypertrophy should prompt the clinician to obtain an overnight polysomnogram to assess for nocturnal hypoxemia and sleep-disordered breathing.

## Markers of Inflammation

SCD is a proinflammatory state with baseline increased levels of serum inflammatory markers, which steeply increase during VOE and ACS. It is likely that this heightened level of baseline inflammation compared to normal individuals plays a role in the pathogenesis of pulmonary and vascular disease. Airway obstruction and hyper-responsiveness, which is seen with increased frequency in patients with SCD compared to the general population, perhaps represents a distinct pulmonary disease entity resulting, in part, from underlying hemolysis. In individuals with genetic/familial asthma, the pulmonary disease is plausibly worse due to disease-modifying effects. More studies are needed to explore this hypothesis. Serum markers, such as reticulocyte count and LDH, should be used to identify and aggressively treat individuals with an increased hemolytic rate as these individuals are at higher risk of complications[66,67]. The role of FeNO measurement is not entirely clear in SCD at this time and further studies are needed to determine the potential of FeNO as a surrogate marker of pulmonary disease in this population.

## Pulmonary Function Tests (PFT)

Abnormal lung function is commonly seen in SCD. Obstructive changes are typically seen in young children[68,75,76], whereas a restrictive pattern dominates in older children and adults with SCD[77]. Klings et al. also reported a high prevalence of low-diffusing capacity in adults with SCD[77]. Koumbourlis et al. reported abnormal lung function in the form of lower airway obstruction even in infants with SCD, thus suggesting that pulmonary involvement begins early on[78]. It is thought that chronic pulmonary inflammation and recurrent episodes of ACS contribute to fibrotic changes in the lungs, leading to restrictive lung disease. It is not clear if children who have an obstructive pattern of PFT and asthma phenotype develop a restrictive pattern over time. More longitudinal studies are needed to evaluate this. A recent study by Knight-Madden et al. demonstrated that adults with recurrent ACS episodes had lower measures of total lung capacity (TLC) and forced vital capacity (FVC) compared to those with one or no episodes of ACS[76]. MacLean et al. showed that there is significant decline in spirometric lung volumes across childhood in SCD. The average decline for forced expiratory volume in 1 sec (FEV1) and TLC is 2.93 and 2.15% predicted per year for males and 2.95 and 2.43% predicted per year for females, which is similar to children with cystic fibrosis and more than those with typical asthma[79]. Early evidence suggests some protection from decline in lung function in children with SCD after successful bone marrow transplant[80].

It is important to monitor PFT on a routine basis in patients with SCD. We propose performing spirometry one to two times per year to evaluate airway obstruction and response to therapy as is routinely done in comprehensive asthma clinics. Full PFT, including plethysmography and diffusion capacity measurements corrected for hemoglobin, should be done once a year. For patients with normal lung function at baseline, but suspicion of AHR, the methacholine challenge test may be considered. However, since a case report of hospitalization for painful VOE following a methacholine challenge study was reported by Knight-Perry et al.[81], the risk-benefit ratio should be strongly considered before doing the methacholine challenge in patients with SCD. Given an association between asthma/obstructive lung disease and pulmonary hypertension[16], and the difficulty in separating pulmonary symptoms of asthma vs. pulmonary hypertension, an annual screening Doppler echocardiography is also recommended in these subjects.

## MANAGEMENT OF ASTHMA IN SCD

There are no consensus guidelines for the management of asthma in SCD at this time and controlled trials of asthma therapy are lacking in this population. In the absence of any evidence-based clinical guidelines, it is recommended that asthma in SCD is managed based on established NAEPP guidelines for the treatment of asthma[17] (Table 1)[68].

**TABLE 1**
**SCD Asthma Management[68]**

1. Treat asthma based on National Institutes of Health (NIH) asthma guidelines[17,93,94,95]: inhaled bronchodilators as rescue medication for respiratory symptoms (nebulized albuterol, ± ipratropium bromide) and corticosteroids for moderate/severe exacerbations. Utilize oral prednisone with slow taper at 1–2 mg/kg/day. A 5-day burst may be insufficient and a slower taper over 2 weeks may be indicated. Case reports of rebound pain and ACS have been described after corticosteroids are withdrawn[85,96,97,98]; however, they should NOT be withheld during an asthma exacerbation. Use of steroids in SCD is a topic of current interest and investigation; however, no interventional studies of SCD and asthma have been performed to date. Pulmonologists familiar with SCD universally recommend treating asthma per the NIH guidelines in SCD.
2. Liberal use of inhaled steroids (asthma controller medication) for persistent asthma symptoms; consider other controller medications, such as leukotriene inhibitors (montelukast).
3. Consult pulmonary or hematology specialist when placing SCD patient on corticosteroids.
4. Hospital admission for all asthma exacerbations requiring corticosteroids.
5. Low threshold to admit mild asthma exacerbations given associated complications.
6. Close monitoring and follow-up is essential.
7. Pulmonary function testing as an outpatient should be followed annually.
8. Screen SCD patients with asthma for pulmonary hypertension by Doppler echocardiography annually.

### Anti-Inflammatory Therapy

For persistent asthma, daily inhaled steroid therapy should be considered as the first choice for asthma control. Leukotriene receptor antagonists (LTRA), such as montelukast, may be considered as an adjuvant therapy. Leukotriene inhibitors may be a promising therapy in SCD given evidence of elevated urinary levels of LTE4 at baseline and during pain crises or ACS[48]. Studies evaluating the efficacy of leukotriene inhibitors in decreasing morbidity in patients with SCD are needed.

Long-acting β-agonists (LABA) may be considered for patients with persistent asthma symptoms uncontrolled with inhaled steroids alone or after addition of leukotriene inhibitors. However, caution should be used in prescribing LABA (salmeterol, formeterol-containing products) in general due to an increased mortality risk identified in African Americans[82], in addition to the high frequency of prolonged QTc reported in SCD[83]. A screening ECG should be considered prior to prescribing LABA in this population given a high prevalence of prolonged QTc interval in SCD patients[83]. As a general rule, patients with SCD and asthma should be followed by a pulmonologist because of the high risk of complications and mortality associated with this comorbidity.

For management of an acute asthma exacerbation, first and foremost importance should be placed on treating hypoxemia to prevent the sickling of red blood cells. Short-acting β-agonists (SABA) should be used liberally, but with perhaps careful monitoring given the high prevalence of prolonged QTc in SCD[84]. Systemic corticosteroids should be used for moderate/severe exacerbations. Due to similarities in presentation and clinical features of ACS and acute asthma exacerbation, systemic steroids should be considered in patients with a personal or familial history of asthma, wheezing on exam, and a good response to bronchodilators. Retrospective reviews and case reports of rebound pain and ACS have been described after corticosteroids are withdrawn, suggesting that these patients need to be closely monitored[85,86]. However, there are studies that document the benefits of systemic steroids for pain and ACS, and a more recent study demonstrates that corticosteroids can be safely used in SCD patients[87]. A retrospective study of 53 patients with SCD showed no significant increase in readmission rate after using prednisone 2 mg/kg/day for 5 days for moderate to severe ACS[88]. It is likely that the potent anti-inflammatory effects of these agents lead to clinical improvement, but abrupt withdrawal triggers rebound inflammation in some patients. Therefore, steroids in tapering doses should be considered.

Frequent use of systemic corticosteroids for asthma exacerbation, high doses of inhaled steroids, and chronic oral steroids for poorly controlled, severe, persistent asthma pose a risk of systemic side effects in patients with SCD just as they do in the general population. In patients with SCD, long-term use of systemic steroids may be associated with an increased incidence of pain crises, ACS, renal infarction, and avascular necrosis of the hip[89]. It is thought that the increase in white blood cell count typically induced by corticosteroids may contribute to an increased risk of adverse events in a patient with SCD. Therefore, chronic systemic steroid therapy for poorly controlled, severe, persistent asthma should be used with caution and such patients should be followed closely by both a pulmonologist and hematologist. To reduce the rate of complications, chronic transfusion therapy should be considered prior to starting chronic systemic steroids[89].

## Other Therapies

Since many complications of SCD result, in part, from an increased hemolytic rate[69], it is logical to think that decreasing hemolysis would improve morbidity and mortality in SCD. Chronic blood transfusions reduce the rate of hemolysis and decrease painful VOE and ACS episodes[90], and may indirectly reduce progression of lung disease, although this association has not yet been studied. Similarly, hydroxyurea has been shown to reduce painful episodes and ACS events[91], and possibly improves chronic hypoxemia[92], thereby reducing pulmonary morbidity in SCD. Novel therapies that target improvement in arginine and NO bioavailability may also have a role in the treatment of pulmonary and vascular complications of SCD. The impact of bone marrow transplant on pulmonary dysfunction deserves further consideration[80].

## CONCLUSION

The high frequency of asthma in this population cannot be attributed to genetic predisposition alone and likely reflects, in part, the contribution of overlapping mechanisms shared between these otherwise distinct disorders. Patients with SCD may potentially be at risk for an asthma-like condition triggered or worsened by hemolysis-driven release of erythrocyte arginase, low NO bioavailability, and subsequent alterations in the arginine metabolome, in addition to classic familial asthma. Regardless of the etiology, hypoxemia induced by bronchoconstriction and inflammation associated with asthma exacerbations will contribute to a cycle of sickling and subsequent complications of SCD. Early recognition and aggressive standard-of-care management of asthma are the keys to prevent serious pulmonary complications and reduce mortality. However, no studies have yet systematically and rigorously characterized the “asthma phenotype” in adults and children with SCD, and data regarding the management of pulmonary complications in SCD are very limited. Clinical trials are needed in order to evaluate the effectiveness of current asthma therapy in patients with SCD and evidence of asthma, while mechanistic studies are needed to delineate the underlying pathophysiology.

## ACKNOWLEDGMENT

This research was supported in part by the FDA grant R01 FD003531-02.
